# Data-driven information extraction and enrichment of molecular profiling data for cancer cell lines

**DOI:** 10.1093/bioadv/vbae045

**Published:** 2024-03-16

**Authors:** Ellery Smith, Rahel Paloots, Dimitris Giagkos, Michael Baudis, Kurt Stockinger

**Affiliations:** Institute for Intelligent Information Systems, Zürich University of Applied Sciences, 8400 Winterthur, Switzerland; Department of Molecular Life Sciences, University of Zürich, 8057 Zürich, Switzerland; Swiss Institute of Bioinformatics, 1015 Lausanne, Switzerland; Infili Technologies, Zografou 15772, Athens, Greece; Department of Molecular Life Sciences, University of Zürich, 8057 Zürich, Switzerland; Swiss Institute of Bioinformatics, 1015 Lausanne, Switzerland; Institute for Intelligent Information Systems, Zürich University of Applied Sciences, 8400 Winterthur, Switzerland

## Abstract

**Motivation:**

With the proliferation of research means and computational methodologies, published biomedical literature is growing exponentially in numbers and volume. Cancer cell lines are frequently used models in biological and medical research that are currently applied for a wide range of purposes, from studies of cellular mechanisms to drug development, which has led to a wealth of related data and publications. Sifting through large quantities of text to gather relevant information on cell lines of interest is tedious and extremely slow when performed by humans. Hence, novel computational information extraction and correlation mechanisms are required to boost meaningful knowledge extraction.

**Results:**

In this work, we present the design, implementation, and application of a novel data extraction and exploration system. This system extracts deep semantic relations between textual entities from scientific literature to enrich existing structured clinical data concerning cancer cell lines. We introduce a new public data exploration portal, which enables automatic linking of genomic copy number variants plots with ranked, related entities such as affected genes. Each relation is accompanied by literature-derived evidences, allowing for deep, yet rapid, literature search, using existing structured data as a springboard.

**Availability and implementation:**

Our system is publicly available on the web at https://cancercelllines.org.

## 1 Introduction

Cancer research is one of the most challenging and promising biomedical areas as reflected in the amount of attention it receives (Cabral *et al.* 2018, Elmore *et al.* 2021, Siegel *et al.* 2022). Cancer cell lines are important models for the study of cancer-related pathophysiological mechanisms as well as for pharmacological development and testing procedures. Cell lines are obtained from patient-derived malignant tissue and are cultivated *in vitro*, potentially in an “immortal” way. Cancer cell lines are supposed to retain most of the genetic properties of the originating cancer (Mirabelli *et al.* 2019), including genomic modifications that are characteristic for the respective disease’s pathology and are absent in normal tissues.

A class of mutations ubiquitous in primary tumors and derived cell lines are genomic *copy number variants* (CNVs) which represent structural genome variations in which genomic segments of varying sizes have been duplicated or deleted from one or both alleles. The set of CNVs observed in a given tumor (“CNV profile”) frequently includes one or multiple changes characteristic for a given tumor type. For instance, while many colorectal carcinomas display duplications of chromosome 13 (Baudis 2007, Lassmann *et al.* 2007), neuroepithelial tumors frequently show small, often biallelic deletions involving the CDKN2A gene locus on the short arm of chromosome 9 (Bostrom *et al.* 2001, Hoischen *et al.* 2008, Rao *et al.* 2010). Recurring CNV events are supposed to be driven by their selective advantage for cancer cells, i.e. recurrently duplicated regions predominately will affect genes favorable for a clonal expansion (“oncogenes”) and, conversely, deleted regions will frequently contain growth-limiting (“tumor-suppressor”) genes (Vogelstein *et al.* 2013).

The collection and comparative analysis of cancer and cancer cell line CNV data is important for the understanding of disease mechanisms as well as the discovery of potential therapeutics. Progenetix (Baudis and Cleary 2001, Huang *et al.* 2021) is a knowledge resource for oncogenomic variants, mainly focusing on representing cancer CNVs. A recent spin-off from the Progenetix resource is *cancercelllines.org—*a database dedicated to genomic variations in cancer cell lines. In addition to CNVs, *cancercelllines.org* also includes information about sequence variations such as single nucleotide variants (SNVs), assembled from the aggregation of genomic analysis data of cell line instances. Currently over 16 000 cell lines from over 400 different cancer diagnoses are represented in this resource.

Natural language processing (NLP) has proven to be a game-changer in the field of clinical information processing for attaining pivotal knowledge in the healthcare domain (Landolsi *et al.* 2022). In fact, numerous studies have been undertaken in exploring indirect relations between drugs, diseases, proteins and genes from unstructured text provided in literature resources. One among many is (Subramanian *et al.* 2020), where the authors systematically design an NLP pipeline for drug repurposing via evidence extraction from PubMed abstracts. Even though such studies exhibit some promising performance, neither ground truth is considered for further relevance evaluation of discovered drug-cancer therapeutic associations nor visualization of results is provided. Additionally, SimText (Macnee *et al.* 2021), a text mining toolset built for visualization of similarities among biomedical entities, manages to extract and display knowledge interconnections from user-selected literature text. However, no quantitative metrics were presented for evaluating the efficiency of the utilized NLP methods.

**Figure 1.**
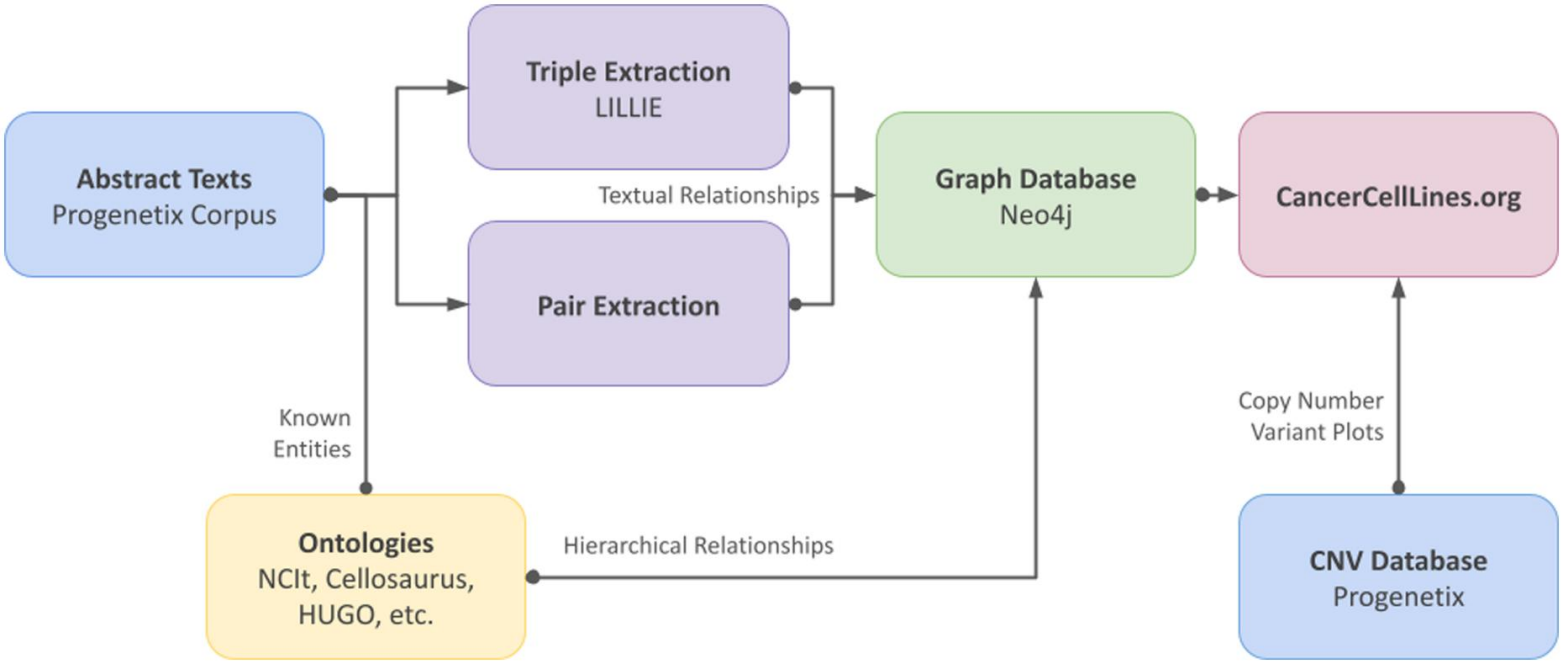
An overview of the architecture of our system, which provides a bridge between unstructured textual corpora and structured clinical data. We first use abstract texts from the Progenetix corpus, along with entity names and synonyms from existing biomedical ontologies such as NCIt and Cellosaurus, to identify textual relational triples using the LILLIE OpenIE system. We then use these triples, along with the relationships from these ontologies, to build a graph database, which is then mapped to existing CNV plots from the Progenetix structured database.

In this article, we study how to use state-of-the-art information extraction algorithms such as LILLIE (Smith *et al.* 2022) to identify known mutated genes and find out which genes are most likely affected in certain CNV regions. As a result, we introduce a novel data exploration system, allowing for the dynamic visualization and exploration of previously orthogonal data models by extracting and enriching information from both structured and unstructured data. An overview of the architecture of our system is shown in Fig. 1. By using the tool, the user will be able to visualize gene information extracted by our algorithm on the CNV profiles of cancer cell lines. The source code for our system is available to the public on GitHub (https://github.com/progenetix/cancercelllines-web).

## 2 Methods

### 2.1 Proposed method

The Progenetix project curates individual cancer Copy Number Aberration (CNA) profiles and associated metadata from published oncogenomic studies and data repositories, which, over the past 22 years, has resulted in the most comprehensive representation of cancer genome CNA profiling data available today (Huang *et al.* 2021). The project consists of both these structured CNA profiles and a manually curated corpus of the associated literature from which these data were derived, but currently the two remain as heterogeneous entities from an automated exploration standpoint. In this article, we propose a novel end-to-end methodology that aims to bridge this gap by combining information extracted from unstructured text (i.e. publication abstracts from PubMed) with structured knowledge resources (i.e. Progenetix and cancercelllines.org) in order to construct an interface for exploratory analysis of positionally mapped genomic variations based on literature evidence.

Our work mainly consists of two parts: (i) fine-tuning LILLIE (Smith *et al.* 2022), a state-of-the-art information extraction tool, in the cancer cell lines context and (ii) development of a portal that serves as the interface for linking various genomic CNV findings with evidence extracted from literature text.

**Figure 2.**
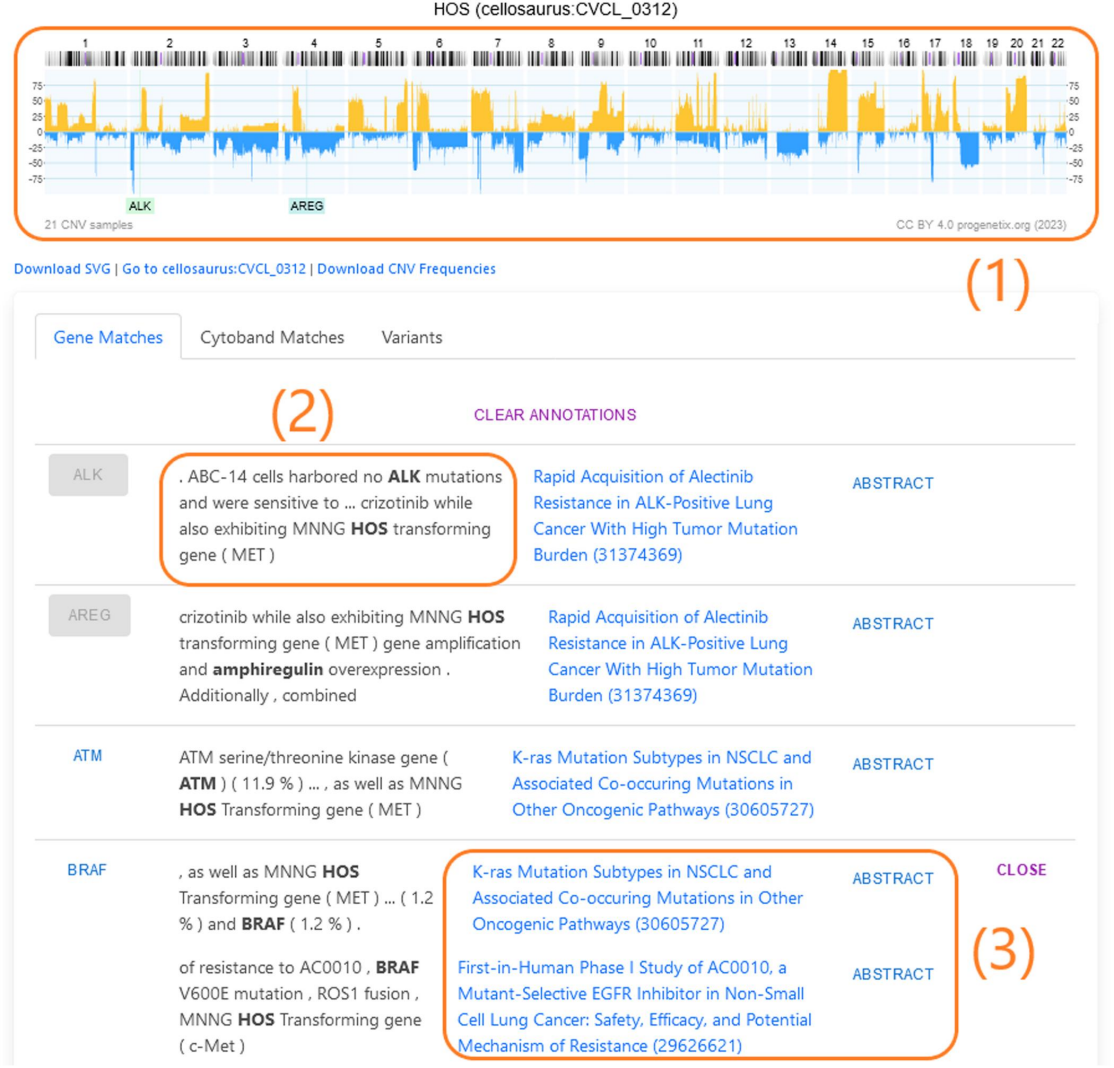
A sample of the results available for the cell line *HOS*, including: (1) associated genomic locations mapped on the copy number variation profile plot (gain CNVs positive values, loss CNVs negative values); (2) evidences for each result; (3) and the relevant abstracts from which the results were derived. The results columns are, from left to right: Gene, Cytoband, or other entity labels; Primary evidence for each abstract (the relevant cell line/entity annotations are marked in bold); Abstract title, and a link to the corresponding PubMed article; Expand/Collapse controls to view detailed information (shown in Fig. 5).

More specifically, we use cell lines as a jumping-off point to provide our literature extraction results. For each cell line, we visualize a corresponding CNV plot, which is annotated by selected extracted genes, and a categorized, ranked list of related entities, as shown in Fig. 2 and on the results page of our system (https://cancercelllines.org/cellline/?id=cellosaurus : CVCL_0312). We provide the most relevant evidence for the given result alongside the title of each paper, allowing the user to easily check the validity of the result, and a toggle to expand each result, revealing the full annotated abstract text, as shown in Fig. 5.

### 2.2 Information extraction from unstructured text

While there are many existing systems which focus on either the topic of biomedical text extraction (Landolsi *et al.* 2022) or the creation of knowledge graphs from text (Xu *et al.* 2020, Franklin *et al.* 2021, Qu and Cui 2021), the *main challenge of our approach was to merge these two concepts* with an existing structured database, such that both can be explored in parallel, and provide complementary information in a streamlined fashion.

Rather than using a known benchmarking dataset for either information extraction or knowledge graph creation, as for example, explored in Mohamed *et al.* (2019), we designed our system using an existing live knowledge base, with a focus on pragmatic data exploration of real-world data, rather than test-set performance.

**Figure 3.**
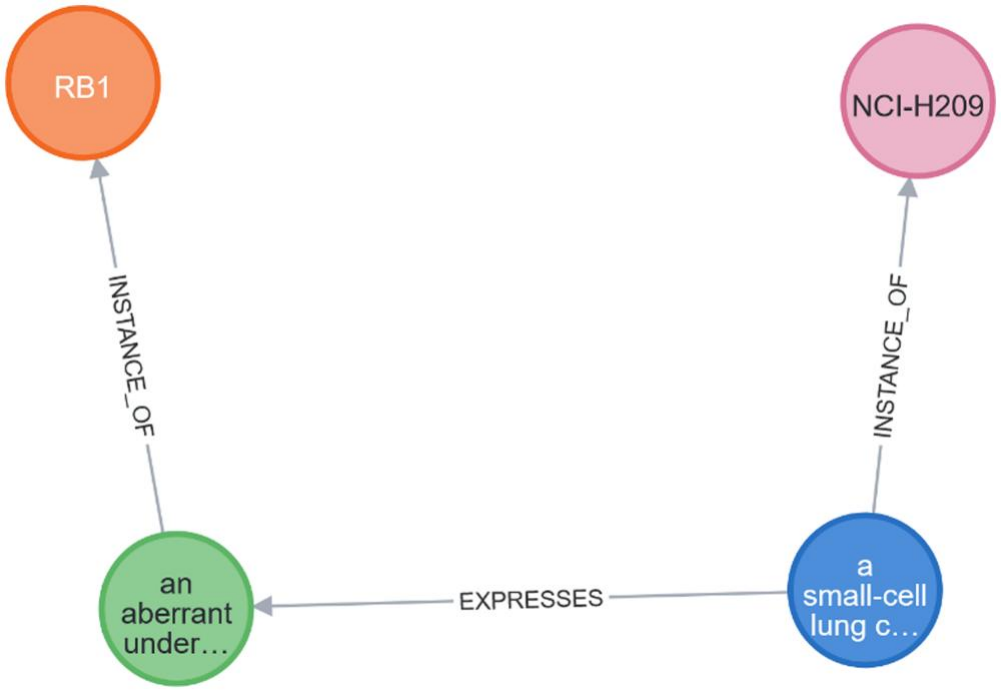
Graph representation of the relationships in the text “*a small-cell lung cancer cell line (NCI-H209) expresses an aberrant underphosphorylated form of the retinoblastoma protein RB1*,” deriving an EXPRESSES relationship between the cell line *NCI-H209* and the gene *RB1*.

Prior work in the development of the LILLIE system (Smith *et al.* 2022), provides a test-case for the concept of applying open information extraction (OpenIE) to database enrichment. When given a piece of unstructured text, subject-predicate-object relational triples are output by LILLIE. The format of these triples is as follows: given the sentence “A small-cell lung cancer cell line (NCI-H209) expresses an aberrant underphosphorylated form of the retinoblastoma protein RB1.,” the following triple is output:

  (RB1 [the retinoblastoma protein RB1];

  EXPRESSES [expresses an aberrant

       underphosphorylated form];

 NCI-H209 [small-cell lung cancer

       cell line (NCI-H209)])

where the subject, *RB1*, predicate, *EXPRESSES* and object, *NCI-H209*, are annotated by their textual context, shown in square brackets. The use of named entities in combination with textual context allows for entities to be matched to structured data, while providing additional contextual information alongside each connection. An example of this is shown in Fig. 3.

While the system was designed for the purposes of OpenIE, which works across a broad range of textual domains, the potential for the application of the same system to a closed-domain task is also demonstrated, along with the viability of the output triples to be integrated to a structured database.

The LILLIE system gives state-of-the-art performance on the most widely-used OpenIE benchmarking datasets, CaRB and Re-OIE16, with F1 scores of 66.4% and 53.9%, respectively, versus 61.7% and 52.7% for OpenIE6 (Kolluru *et al.* 2020a) and 61.7% and 53.5% for IMoJIE (Kolluru *et al.* 2020b), and AUC score improvements of up to 6% over the previous state-of-the-art. Additionally, it includes features for adjusting and fine-tuning the output triples to potentially match any closed-domain task.

The system consists of a *pre-trained learning-based component*, based on open-domain corpora, and a *hand-optimized rule-based component*, which is parameterized to allow fine-tuning of the output rules for a closed-domain task. In Smith *et al.* (2022), it is shown that the adjustment of these rules can produce a customized Information Extractor for the use-case of linking anatomical entities and diseases with gene expressions, which can then be integrated into a relational database.

Here, we expand upon this work by applying a customized version of LILLIE to extract relational triples in the context of cancer cell lines, and integrate these triples into a graph database, allowing existing hierarchical ontologies to be linked with textual relationships in a structured manner, which we describe in detail in the following sections.

#### 2.2.1 Triple extraction

Other recent work in the field of biomedical information extraction focuses on recognizing a predecided set of relations (Patrick *et al.* 2011, Luo *et al.* 2022). However, here we use the OpenIE paradigm (Liu *et al.* 2016) to extract any potential relationship between entities in the text, namely, as natural language subject-predicate-object triples. The use of this model allows a researcher to explore richer and more descriptive relations between entities than if they were mapped to discrete categories, and takes into account the fact that relationships in oncogenomics are often complex and subtle. Thus, OpenIE methods, coupled with domain expertise, was determined to be optimal for this use case.

For this work, we use a customized version of the LILLIE system, for which we tailor the rules in the rule-based component to better suit the context of this task. Since scientific abstracts are typically written in a formal manner, many sentences contain complex conjunctions, and long-distance dependencies over multiple clauses. However, the language used is unambiguous and well-constructed, unlike in open-domain tasks. For example, the sentence “2-O-Methylmagnolol Upregulates the Long Non-Coding RNA, GAS5, and Enhances Apoptosis in Skin Cancer Cells” is complex to parse, but unambiguous in meaning.

As described in Smith *et al.* (2022), it is possible to modify the triple parser and the output format of the LILLIE system to suit the target linguistic profile of the input texts. It is shown that in the context of biomedical paper abstracts, enabling and disabling certain features can produce an increase in entity-matched triples of 46.5% over the unmodified version of LILLIE used in the open-domain context, and an increase of 42% and 41% over OpenIE6 (Kolluru *et al.* 2020a) and IMoJIE (Kolluru *et al.* 2020b), respectively, in the same context. In addition, we found that in this context, due to these linguistic factors, modifying LILLIE to use only the rule-based component produced a higher quality of results based on qualitative analysis of the end-user exploration portal by domain experts.

### 2.3 Entity linking

After running the LILLIE system on the abstracts of all research articles in the Progenetix corpus to produce a set of textual triples for each abstract. Next, we match the subject and object with their corresponding entities in the following ontologies:

- The cancer section of the NCIt thesaurus (Sioutos *et al.* 2007)
- The UBERON anatomical ontology (Mungall *et al.* 2012)
- The Cellosaurus cell-line index (Bairoch 2018)
- Cytogenetic mapping information from Progenetix (Huang *et al.* 2021)
- The HUGO gene nomenclature (Tweedie *et al.* 2021)

Each ontology provides an identifier for a given entity, along with a canonical name and a set of synonyms for each entity. We use dictionary-based methods (Quimbaya *et al.* 2016) to match a triple to its corresponding entities. First, we preprocess the entity names in each ontology. For gene and cell line canonical names, we leave these in their original state without any processing, as even changing the case of a gene (e.g. “STEP” and “step”) can cause ambiguities. Otherwise, we expand each of the synonyms provided by the ontologies into two forms: one which has been lemmatized and tokenized and another which has only been case-normalized. We use these processed ontologies to construct a categorical entity dictionary.

We then represent the text of the subject and object of each triple in three forms: unprocessed, tokenized and lemmatized, and case-normalized. Using our dictionary, we mark each triple as containing an entity when a token overlap occurs with one of the entity forms in our dictionary. This method was chosen to emphasize precision and reduce spurious matches, since in biomedical text extraction, particularly when the domain is narrow, dictionary-based approaches for entity-relation extraction have been shown to give comparable performance to learning-based methods (Landolsi *et al.* 2022), due to the fact that textual biomedical entity references are typically precise, unambiguous and correspond directly with their canonical names.

**Figure 4.**
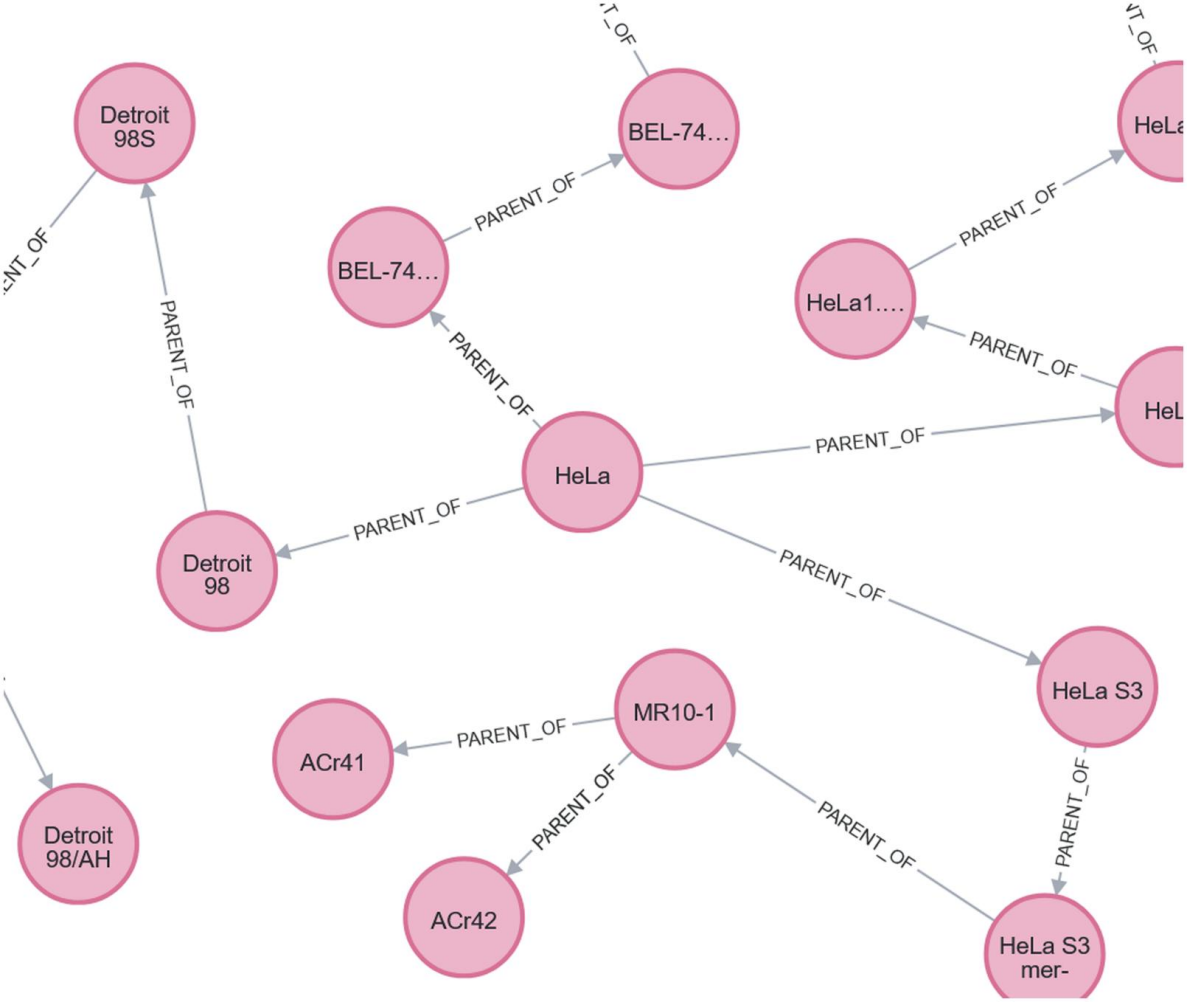
Portion of the cell line hierarchy for *HeLa*, showing the entity itself, and its daughter cell lines. Nodes in the graph are derived from the ontologies (in this case, Cellosaurus), and the edges indicate a “*parent-of*” relationship.

In addition to an entity dictionary, we also use the ontologies described above to construct a hierarchical graph ontology, where entities are nodes, and parent–child relationships are represented by edges in the graph. A sample of this ontology is shown in Fig. 4, which depicts a portion of the resultant subgraph for the cell line *HeLa*, derived from Cellosaurus.

We then place these entity-matched triples in a graph database, as shown in Fig. 3. Unlike in other works on biomedical knowledge graph building (Mohamed *et al.* 2019), we do not infer any relations using the graph itself. Only relations directly implied by the text are present in the graph, as shown in Fig. 3. In contrast, Mohamed *et al.* (2019) attempt to synthesize whether a relationship exists in between two nodes based on existing relationships using a knowledge graph embedding approach. Our aim here is to provide a link between existing evidences (between natural language and structured data), rather than synthesize new knowledge using machine learning methods.

#### 2.3.1 Pair extraction

**Figure 5.**
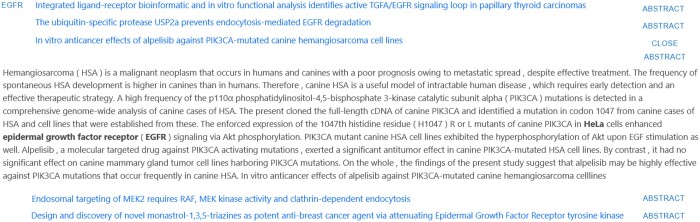
Section of the results demonstrating a relationship between the cell line *HeLa* and the gene *EGFR*, showing the paper title, primary evidence for the result, and, when expanded, the full annotated abstract text.

**Figure 6.**
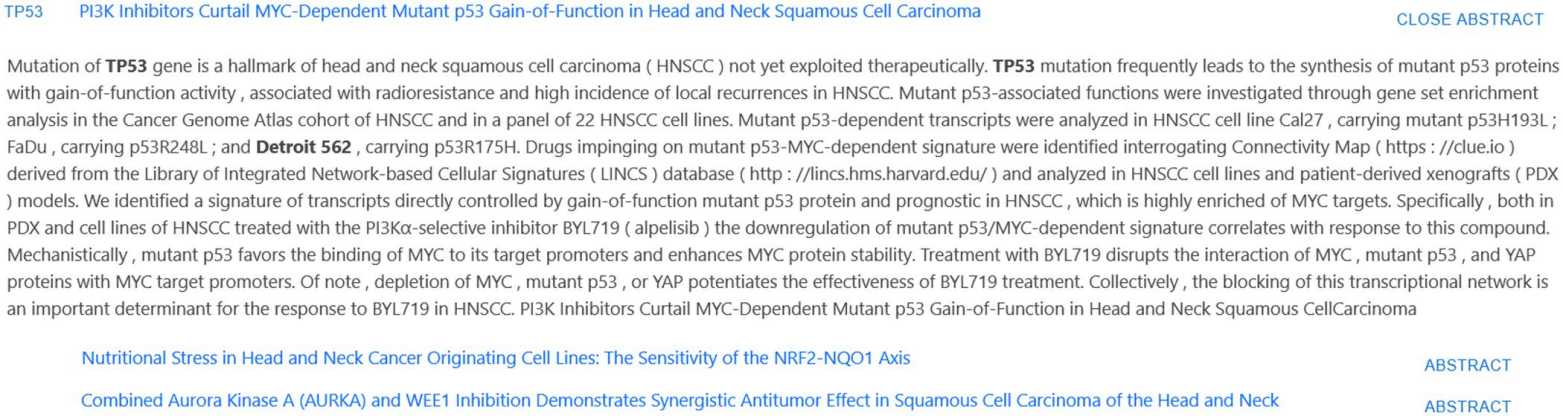
Section of the results demonstrating a relationship between the cell line *Detroit 562* and the gene *TP53*, showing the paper title, primary evidence for the result, and, when expanded, the full annotated abstract text.

While triple extraction can expose deep semantic relations between entities, this approach does not necessarily provide a complete representation of all relationships within the text, as it only extracts predicates that are directly expressed as singular verb phrases. An example of a strong sentential relationship extracted by triples is shown in the abstract in Fig. 5, whereas long-distance relationships, as shown in Fig. 6, are not currently reliably extractable using similar methods. This is a known shortcoming of current information extraction techniques, and recent efforts such as BioRED (Luo *et al.* 2022) have attempted to mitigate this deficiency by providing a corpus of long-distance relations that may span an entire document. However, the BioRED corpus is limited in both the number of relationship annotations and the fact that no specific annotations for cell lines are provided.

Naturally, if two entities are present in the same textual snippet, they are likely related in some manner, though this is not easily represented in the standard subject–predicate–object model. As such, we augment our triple extraction with what we term as *pair extraction*, where we extract subject–object pairs, but leave the relation expressed as a simple numerical quantity. We combine these pair extractions with triples to produce a more representative ranking in the final output.

As such, we *augment our high-precision triples with an additional high-recall method to capture long-distance relations*, using information retrieval techniques such as term distance. We use the following metric to rank relationships between entities:

$$\begin{array}{c} R(D,e_{1},e_{2})=\sum_{P(e_{1})\in D}\sum_{P(e_{2})\in D}{\;\text{log}\;}_{2}^{-1}\left(|P(e_{1})-P(e_{2})|+1\right) \end{array}$$

where *D* is an abstract document, $e_{1}$ and $e_{2}$ are the pair of entities found in the abstract, $R(d,e_{1},e_{2})$ is the relationship score between $e_{1}$ and $e_{2}$ in a given abstract, and $P(e_{n})$ is the token span position of entity $e_{n}$ in the abstract. Essentially, we model the potential relationship between entities by the sum of the inverse logarithms of the token distance between each instance of a given entity. If an entity pair is also marked as being part of a triple, we set $R(D,e_{1},e_{2})+=1$ for each occurrence of a triple, as this represents a definitive semantic connection. Thus, when combined with our semantic triple extraction, we can, given a pair of entities of interest, such as a gene and a cell line, produce a ranked list of abstracts, ordered by the strength of their relation, using this metric. In our portal, we then produce a ranked list of genes for each cell line based on the total score for that pair across all the literature in our database.

An example of such a pair extraction is shown in Fig. 6, where a complex relationship between *Detroit 562* and *TP53* is extracted as a pair relationship, but cannot be represented as a semantic triple using even current state-of-the-art information extraction techniques. We capture this relationship using our augmented approach, but such an abstract would have a lower weighting than one containing a triple-based relationship, due to it being a weaker inference. However, by highlighting a potential relationship to the user, a researcher can make a judgement on its relevance, which bridges the gap while current information extraction techniques are insufficiently mature enough to perform such tasks alone. Conversely, in Fig. 5, the cell line *HeLa* is explicitly linked with *EGFR* through a triple relation, and is ranked higher due to the known presence of a strong semantic correlation.

## 3 Results

In this section, we will first apply our information extraction system for analyzing various cancer types. Afterwards, we will evaluate the performance of our automatic information extraction pipelines. In particular, we want to address the following two research questions:

- *Research question 1: How well does our information extraction pipeline work for studying cancer cell lines and for exploring potentially new information?*
- *Research question 2: What is the performance of our automatic information extraction algorithm for combining structured and unstructured data, i.e. from a database for cancer cell lines and research abstracts from PubMed?*

### 3.1 Example use cases

To validate the efficacy of our approach, we analyzed the results of our novel information extraction pipeline and how the extracted data corresponds to cell line CNV profiles. We will now illustrate how to analyze two different cancer types using our approach with the help of two example use cases.

#### 3.1.1 Head and neck squamous cell carcinomas—cell line Detroit 562

**Figure 7.**
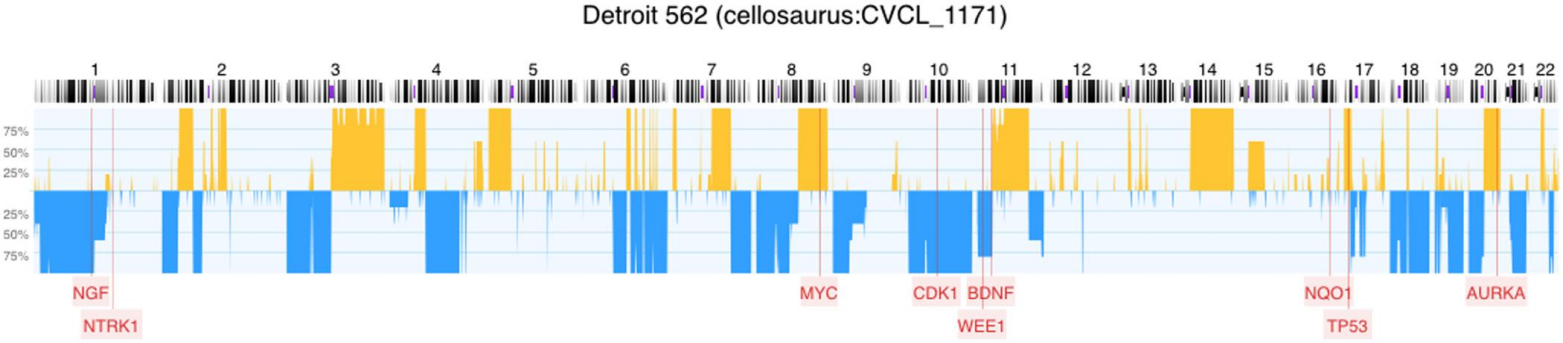
CNV frequency profile of 5 instances of the cancer cell line Detroit 562 annotated with enriched gene information from our information extraction pipeline. Copy number gains are shown above the median and deletions below the median. Of note, a few of the regional CNVs deviate from the 100% expected for stable clonal propagation due to some genomic instability and possible variation in the fidelity of individual profiling experiments. Mapping positions of genes of interest are shown as labels below the profile.

Figure 7 depicts the CNV profile for Detroit 562—a pharyngeal squamous cell carcinoma cell line (NCIT code C102872). Pharyngeal squamous cell carcinoma is a part of head and neck squamous cell carcinomas, often related to smokers. The results of our information extraction pipeline for genes AURKA and WEE1 claim that these genes are highly expressed and down-regulated (these results can be reconstructed here: https://cancercelllines.org/cellline/?id=cellosaurus : CVCL_1171), respectively, in cancers, see Lee *et al.* (2019). This information is confirmed on the CNV profile where AURKA is duplicated and WEE1 is deleted. Similarly, MYC gene is brought forward as a possible target due to high expression and the region is duplicated on the CNV profile as well.

Figure 7 also indicates TP53, a tumor-suppressor gene involved in the control of cell division located on the short arm of chromosome 17. Due to its inhibitory role on cellular expansion, it is a frequent target of genomic deletions in a variety of cancers. However, TP53 can also acquire gain-of-function mutations that contribute e.g. to radioresistance, thus, explaining the duplication in this region in the case of a mutant allele (Ganci *et al.* 2020). Conversely, NGF—a gene, i.e. reported to be expressed in Detroit 562, exhibits allelic deletion in our CNV data (Dudás *et al.* 2018), points toward alternative mechanisms responsible for its transcriptional activation.

#### 3.1.2 Breast carcinomas—cell line MDA-MB-453

**Figure 8.**
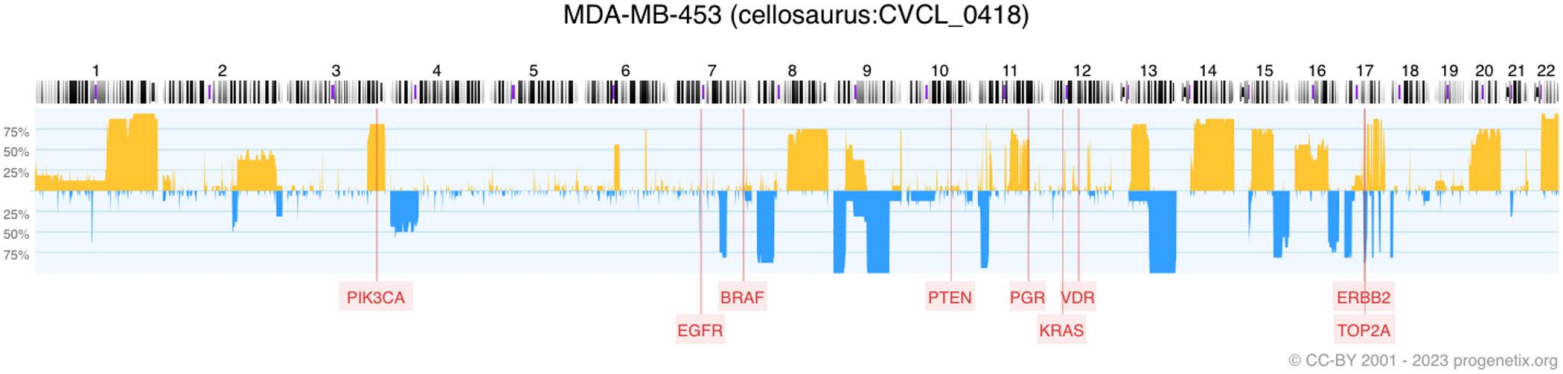
CNV frequency profile of 16 breast cancer cell line MDA-MB-453 samples, annotated with enriched gene information from our information extraction pipeline. Copy number gains are shown above the median and deletions below the median. Mapping positions of genes of interest are shown as labels below the profile.

Breast cancer is the most common cancer type in women, affecting more than 250 000 women in the US alone, see (Siegel *et al.* 2017). In breast cancer, several clinicopathological parameters have been recognized. One of the rare but clinically especially aggressive variants is the “triple-negative” subtype, i.e. where the tumor cells do not express 3 receptors commonly targeted in hormonal and immunotherapy: estrogen receptor, progesterone receptor, and ERBB2 (HER2) receptor. Cell line MDA-MB-453 is a breast cancer cell commonly used to represent the triple-negative expression profile (https://www.cellosaurus.org/CVCL_0418). However, using our information extraction pipeline, we could match this cell line to a publication that claimed its expression of ERBB2 (these results can be reconstructed here: https://cancercelllines.org/cellline/?id=cellosaurus : CVCL_0419), see Santra *et al.* (2017). Indeed, in our CNV data from 16 instances of MDA-MB-453 we can observe genomic duplications involving the ERBB2 locus on 17q (see Fig. 8).

While another paper claims PTEN to be expressed in MDA-MB-453 (Singh *et al.* 2011), the CNV profile does not indicate a genomic duplication event as causative and therefore indicating transcriptional deregulation. We also matched this cell line to 2 papers where mutation in KRAS was confirmed by our SNV data. Moreover, the expression of PIK3CA was confirmed by the duplication on the CNV profile as well as the mutation of the same gene was detected in the SNV data, see (Patra *et al.* 2017).

Thus, to answer *Research Question 1*: We show here that our novel information extraction feature facilitates further research into cancer cell lines. We were able to prove some known gene expression levels for cell lines Detroit 562 and for MDA-MB-453. Moreover, we could discover some new or conflicting information about some other genes.

More insights about how to reconstruct the exploration of these use cases with our system can be found at https://docs.cancercelllines.org/literature-data/.

### 3.2 Information extraction

After applying our information extraction pipeline for studying various cancer types, we will now evaluate the performance in terms of accuracy and number of relevantly linked triples.

#### 3.2.1 Data exploration

The Progenetix and cancercelllines.org resources provide PubMed identifiers for articles with a direct relation to genomic analyses in cancer cell lines. Crawling the PubMed database from these identifiers resulted in a corpus of 52 412 textual abstracts, which were used by our system to generate our graph database. As shown in Table 1, we find 770 230 total entity matches, leading to a total of 12 139 distinct nodes in our graph.

**Table 1.** Number of input abstracts, the number of matched entities found by our system, along with the number of cell lines extracted, and the number of unique relations per cell line.

Number of abstracts	52 412
Total entity matches	770 230
Unique entity matches	12 139
Unique cell lines	1411
Abstracts per cell line	6.09
Linked entities per cell line	53.609

#### 3.2.2 Information extraction performance

As there does not currently exist a benchmark for evaluating relationships between genes and cell lines, we adapt a subset of the BioRED benchmark (Luo *et al.* 2022) to assess the performance of our system. The BioRED benchmark provides annotations for two types of evaluation: entity spans for NER (Named Entity Recognition) and entity–relationship pairs. For the NER task, annotations are provided for both Genes and Cell Lines, which fit the needs of this task. However, while this corpus includes relationship annotations for certain entity types (e.g. Gene–Gene and Gene–Chemical), it does not include Gene–CellLine relationships, which is the target of our platform.

To account for this, we adapt the BioRED annotations as follows: we assume that, for a given abstract, discussion of a given gene and cell line in the same passage implies a causal relationship between the two. While this may introduce false-positives, our exploration platform is designed to extract all potential relations between genes and cell lines, and then allows a domain expert to select those that they believe to be relevant. As such, this adaption of the test corpus represents a viable evaluation metric of the goals of our system, when combined with our qualitative analysis in Section Results.

Based on this, we generate a set of Gene–CellLine relationship pairs for each paper as follows: if both a gene and cell line are annotated as entities in the same abstract, we additionally annotate that paper with a relationship pair for these entities. We then perform the Entity Pair evaluation as described in Luo *et al.* (2022). Our system achieves an F1 score of 74.2% on our adapted BioRED test set for Gene-CellLine pairs. On the original BioRED corpus, BioBERT and PubMedBERT achieve scores between 58.8% and 58.1%, respectively, (for Chemical-Variant relationships) and 78.5% and 78.1% (for Gene-Gene relationships) on the Entity Pair test. We believe that these results, in combination with Table 1 answers *Research Question 2* posed at the beginning of this section.

## 4 Discussion

Starting from a domain specific resource for curated genomic and associated data in cancer cell lines we extended its “classical” online database paradigm toward a knowledge exploration resource through the implementation of our novel literature information extraction algorithms. This change enables researchers to use the existing data—such as annotated genomic variations, visual indication of structural variation events and disease-related annotations—to gain context specific insights into molecular mechanisms through exploration of the added literature-derived information either directly or to prioritize follow-up analyses. We could show that the extracted results can easily be related to the resource’s hallmark CNV profiles and this combination opens possibilities for knowledge expansion, including the critical evaluation of pre-existing annotations which may be affected by the fast-mutating nature of cancer cell lines. In our information extraction implementation, we have shown that an interactive bimodal exploration model can be achieved in a streamlined manner, even if one data source comprises unstructured information.

Ubiquitous application of high-throughput molecular analyses as well as their interpretation in an ever-increasing amount of publications drive a “data deluge” in biomedical research. Our work demonstrates an application of information extraction techniques to add a knowledge exploration dimension to a genomic data resource. By doing so, we provide a tool to increase the speed and depth of scientific research using computational linguistic methods.

For this work, we provide an evaluation on an adapted version of the BioRED corpus [and benchmarking of our information extractor is provided in Smith *et al.* (2022)]. In addition, we provide a qualitative analysis in Section Results, demonstrating that our system can be applied on real-world data to discover new knowledge, and we envision our system as a tool to dynamically discover novel data in tandem with a domain expert.

## Data Availability

The data underlying this article are available at https://github.com/progenetix/cancercelllines-web and https://pubmed.ncbi.nlm.nih.gov/.
